# Urbanisation and sexual health: understanding bisexually active men in Hanoi, Vietnam

**DOI:** 10.1080/21642850.2014.913488

**Published:** 2014-05-05

**Authors:** Peter Higgs, Siobhan Reddel, Hanh Van Pham, Khoat Van Dang, Margaret Hellard

**Affiliations:** ^a^ Faculty of Health Sciences, National Drug Research Institute, Curtin University, Suite 6, 19–35 Gertrude Street, Fitzroy 3065, Melbourne, Australia; ^b^ Centre for Population Health, The Burnet Institute, Melbourne, Australia; ^c^ Vietnamese Community Mobilisation Centre for HIV/AIDS Control, Hanoi, Vietnam

**Keywords:** HIV/AIDS, gay, lesbian, bisexual and transgender, translational research, Hanoi, focus group discussion

## Abstract

*Background*: Men who have sex with men (MSM) in Vietnam are receiving increased attention in recognition of their high-risk behaviours and potential for human immunodeficiency virus (HIV) infection and transmission. Due to societal pressures, many MSM in Vietnam are also bisexually active, which ultimately increases the transmission risks beyond the MSM population. Evidence is emerging that indicates a greater proportion of women in Asia with low-risk sexual activities are contracting HIV from their male partners who have become HIV infected through male–male sex. *Methodology*: Fourteen focus group discussions exploring sexual and social networks were conducted in Hanoi between July 2010 and September 2010. A total of 96 individuals participated in these sessions. *Findings*: A risk environment approach was used to analyse the focus group themes of social stigma and marriage, sex with other men in closed settings and transactional sex in Hanoi, an increasingly urbanising and westernising city. *Implications*: Despite limited evidence globally that bisexual men act as a bridge for sexually transmitted diseases, there is particular concern in Vietnam about this potential risk. HIV rates amongst MSM are rapidly rising and there are reports of women contracting HIV from their male partners who are bisexually active.

## Background

Asia is considered to have one of the most diverse human immunodeficiency virus (HIV) epidemics in the world, and there is increased concern about emerging or newly identified HIV epidemics among men who have sex with men (MSM) in the region (van Griensven & de Lind van Wijngaarden, 2010; UNAIDS, 2008). Within the Greater Mekong Sub-Region (GMS),^1^ as with other parts of Asia, HIV epidemics among MSM are better understood in some countries such as Thailand than in others like Vietnam, where epidemics among MSM are more recently being documented (García, Meyer, & Ward, 2012). In the GMS, most HIV prevalence estimates among MSM report steadily climbing rates. Current areas with lower prevalence estimates include 5.6% (Vientiane), 8.7% (Phnom Penh) and 13.2% (Yunnan, China). Higher rates have been observed in Myanmar (28.8%) and Thailand (31–35%) (van Griensven et al., 2010; Jia et al., 2010; Lowe, 2011; National AIDS Programme, 2009).

HIV prevalence among MSM in Bangkok increased from 3% in 1990 to 17% in 2003 to 28% in 2005 to 31–35% in 2009 (van Griensven & de Lind van Wijngaarden, 2010; Sopheab, Morineau, Neal, & Chhorvann, 2008). Similarly, recent data in Vietnam indicate rapid increases in HIV prevalence in this population, from 5.3% to 14.8% over the period 2006–2009 in Ho Chi Minh City and from 9.4% to 20% between 2006 and 2010 in the capital Hanoi (García et al., 2012; Lowe, 2011; Ministry of Health, 2011; National Committee for AIDS Drugs and Prostitution Prevention and Control, 2012). UNAIDS (2008) predict that by 2020 around half (46%) of new infections in Asia could be amongst MSM, up from 13% in 2008. Vietnamese data also indicate that MSM who inject drugs have rates of HIV up to five times that of non-injecting MSM (Mesquita et al., 2012).

### MSM and the approach to HIV prevention in Vietnam

Until recently Vietnamese government initiatives and media responses have focussed on the HIV epidemic being concentrated in, and driven by, people who inject drugs (PWID) and female sex workers (Sarraf, 2009). HIV sentinel surveillance for PWID and female sex workers was introduced during the early response to the epidemic, but only since 2009 have MSM been included in the surveillance system (García et al., 2012; Nguyen, Nguyen, Le, & Detels, 2008; Viet Nam Administration of HIV/AIDS Control (VAAC), 2009; Vu, Mulvey, Baldwin, & Nguyen, 2011).

MSM were first listed as a high-risk group for HIV/AIDS in 2006 with the revised legal document (decree No 108/2007/NĐ-CP) specifying the implementation of harm-reduction initiatives including condom distribution for MSM (Sarraf, 2009). The Vietnamese government approach reflects the fact that there has been minimal social recognition of male homosexuality in Vietnam in the past. With encouragement and financial support from partner states there have been increased initiatives targeting MSM in Vietnam since mid-2000s; but these continue to be limited, with no specific interventions in the 2009 National Strategy on HIV Prevention and Control (Murphy, Bok, & Phuong, 2009; United Nations, 2010).

The lack of traditional social and political recognition of homosexuality in Vietnam is the likely reason why there are no published population-based data on the proportion of sexually active men in Vietnam who have ever had sex with men. Large population surveys in some Asian countries have found that sex between men is reported more commonly than in most Western countries. Surveys in Australia, the UK and the USA have found that 5–7% of men report ever having sex with a man and 2–3% report that they are primarily sexually attracted to men (Laumann, Gagnon, Michael, & Michaels, 1994; Smith, Rissel, Richters, Grulich, & de Visser, 2003; Wellings, Field, Johnson, & Wadsworth, 1994). By contrast, large sample surveys of 21-year-old Thai military conscripts in the early 1990s found that 17% reported same-sex experiences (Tantirattanong & Kladsawat, 2008). It has been estimated that up to 18% of men in Asia have ‘same-sex relations at some point in their life’ (UNAIDS, 2007, p. 7). To date there is no specific data on the proportion of men who engage in homosexual behaviour in Vietnam, though over 35,000 MSM are estimated to be living in Hanoi (García et al., 2012).

The current estimates of overall population prevalence of HIV in Vietnam are around 0.3–0.53% (Joint United Nations Programme on HIV/AIDS, 2010). Overall sexual transmission of HIV appears to be increasing and injecting drug use transmission decreasing, although the risk factors for over one-third of acquired infections are unknown or unreported (Colby, Minh, & Toan, 2008; United Nations, 2010). Intimate partner transmission to females from HIV positive men is also posited to account for the reducing ratio of HIV prevalence between males and females: in 2008 males were around three times as likely to have HIV as females, but it is predicted that by 2012 this ratio will drop to 2.6 (United Nations, 2010). Notably, official accounts of transmission do not mention the possibility of HIV transmission to women by bisexually active men.

The 2009 Integrated Biological and Behavioural Surveillance Survey (IBBS) reported that 66.5% of MSM reported condom use the last time they had anal sex with a male partner, as opposed to 77.7% of female sex workers reporting condom use with their last sexual partner (Ministry of Health, 2011). Some authors have suggested that the reasons for lower consistent condom use amongst MSM in Vietnam stem from the non-inclusion of homosexual sex as a risk factor for HIV, leading to falsely low perceptions of HIV risk amongst this population (Sarraf, 2009). Hence whilst general knowledge of HIV is typically high among MSM, many still report not knowing how to act on this knowledge to protect themselves from HIV (Colby, 2003; Toan, Colby, & Minh, 2005). Furthermore, low percentages of MSM rate their HIV risk to be high despite their low condom use (Colby, Cao, & Doussantousse, 2004; Colby et al., 2008).

### Bisexually active men

The 2010 World Health Organization South-East Asia report on HIV/AIDS among MSM suggests that significant proportions also report sex with female partners: in India 12–69% of MSM also reported sex with female partners in the preceding six months and in Timor Leste 94% in the preceding 12 months (World Health Organization, 2010). A 2007 study in Lao People's Democratic Republic (PDR) of men who reported sex with a man within the previous six months found that 57% of the participants had also ever had sex with a woman (Sheridan et al., 2009). Of the 5.6% of men in the Lao study who tested HIV-positive almost half (46.7%) reported sex with a woman within the previous three months.

Hence a significant number of women, mostly married and generally considered at ‘low-risk’, may be at risk or become infected with HIV by their spouses. It has been estimated that by 2020, one-fifth of HIV infections will be among women infected by their spouses, of whom a proportion will have a history of male-to-male sex (Commission on AIDS in Asia, 2008).

Preventing HIV infection among married women starts with the prevention of HIV infection in their partners. HIV interventions which aim to address rising infections among MSM in Asia are currently inadequate due to limited coverage and lack of investment in scaling up services. Where services are provided uptake is generally high, however most programmes do not target men who have female partners (Commission on AIDS in Asia, 2008; Phillips et al., 2010).

In order to understand the sexual relationships and practices of bisexual men, it is essential to understand their beliefs, decision-making processes and attitudes towards HIV risk-taking. It is also important to understand the environments in which risk takes place. One way of better understanding this is through what Rhodes has coined the ‘risk environment’ framework. This is one way of being able to understand risk-taking beyond personal health beliefs and conscious individual risk assessments (Rhodes, 2002). More in-depth comprehension of the nuances of risk-taking and the spaces in which risk occurs is required. Further, how our understanding of this can be used to develop and target appropriate HIV prevention interventions will enhance HIV programming in Vietnam (Le, 2004).

## Aims and objectives

This paper reports on the formative qualitative component of a study which explored the context in which sexual interactions of bisexually active men occur (in terms of *meeting* partners, *negotiation* of sex, and *where* the sex act occurs) and how place and time impact on the choice of partner (the intersection of *why* and *where*).

## Methods

This work sits within a larger study that examined the social and sexual networks of bisexually active men in Hanoi, Vietnam. Participants were recruited through the social networks of peer educators and invited to attend a focus group discussion at venues where men who have sex with other men are known to congregate. A range of qualitative research techniques were used; trained facilitators conducted the focus group discussions and led the pair-wise ranking and mapping exercise. The focus group included questions which aimed to capture participant understandings of attitudes to bisexual behaviour, motivations for sex and how this differed across genders, the space and location of sexual encounters and how this differed across genders, condom use and the influence of alcohol or other drugs. Data were translated from Vietnamese into English for thematic analysis to identify dominant themes related to the gender of sexual partners of participants.

Eligibility for participation in focus groups included men living in Hanoi who reported sex **(**oral, anal and/or vaginal intercourse that resulted in ejaculation) with both men and women in the previous 12 months. Informed consent was obtained verbally from participants at the start of the focus group discussion.

The study received ethics approval from The Alfred Hospital Ethics Committee (Melbourne, Australia), the Australian National University Ethics Committee (Canberra, Australia) and the Hanoi School of Public Health (Hanoi, Vietnam).

## Results

Fourteen peer-led focus groups were held with 96 men between August and November 2010. The groups had between five and 11 participants recruited by peer educators and were held at spaces where MSM are known to congregate. There were two specific focus groups for men who inject drugs and also have sex with other men. The age range of participants was 18–47 years.

### Kinsey Scale

**Figure 1.**
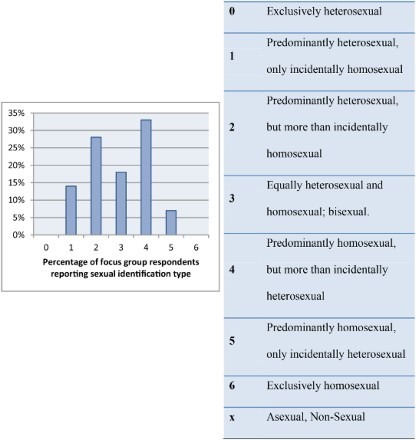
Sexual identification of male participants (Kinsey Scale).

As outlined in the methods, all participants reported a sexual history with both men and women. Each participant was asked to self-rank his perception of sexual behaviour in relation to a seven point Kinsey Scale. Results indicated that none of the participants in the focus groups identified as exclusively homosexual or heterosexual, however well over a third of participants identified as predominantly heterosexual and similarly another third as predominantly homosexual. Eighteen per cent of these bisexually active men identified themselves as bisexual. These proportions are shown in Figure 1.

## Pairwise ranking and place

**Figure 2.**
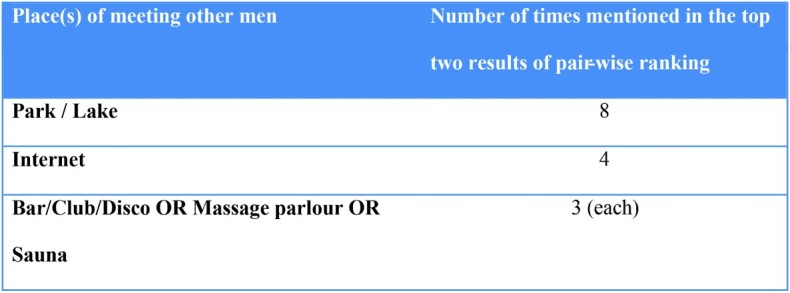
Places to meet other men.

**Figure 3.**
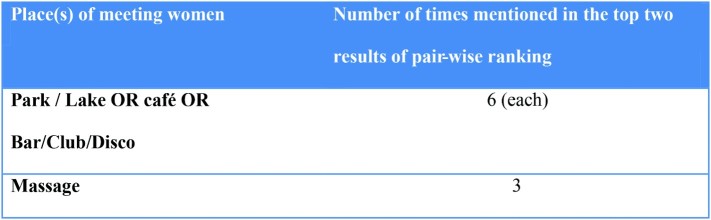
Places to meet women.

Participants were asked to compare the places that they were most likely to go to meet either men or women with the purpose of having sex. These results were summed to assess the most common places of meeting. The results for whether the place was ever mentioned by a group to be in the top two most likely places that men met men and women are presented in Figures 2 and 3, respectively. Public spaces such as parks and lakes featured prominently in meeting both men and women and the internet was the second most likely method for meeting men.

## Key findings

In reviewing the sexual histories recounted during the focus groups, several recurring themes about bisexual experiences were highlighted. First, there was considerable focus on the pressures of traditional culture and gender roles, including marriage, and the consequent shame and stigma associated with male–male sexual relationships (Nguyen et al., 2008; Sarraf, 2009). As the following two narratives explain:

When I was 28 years old, I began to think about marriage to maintain my family name, although I'm not attracted to women. One year ago, I got married. Now I have a 9-month-old child and my wife does not know about my sexuality, partly because she lives in another province and I work here [Hanoi]. After marriage, we committed not to intervene in the private issues of each other…

There are many men having sex with women while they like men because they want to avoid judgements of the wider society…

Without wanting to generalise, it is our view that these narratives reflect the societal prejudice the male participants felt in relation to the Vietnamese expectation of men to get married and have a family. The theme of shame and stigma comes with the pressure this brings to bear on homosexually active men in Vietnam.

Another encompassing and recurring theme of the focus groups was that early homosexual experiences were common in the setting of closed communities such as during compulsory military service and in university dormitories. This was described by an older participant in our focus groups as he reminisced about his time as a soldier.

In 1960s and 1970s, homosexuality was not as common in Vietnam as it is today. I was born and grew up in Hanoi and when I was 18 years old, I joined the army to do my duty for my country and my battalion settled in Ha Giang province. One winter night, I and my colleague had sex due to lack of warmness and to help avoid the harsh weather. After that, we were very shy whenever saw each other. But it continued many times when we would guard together. After leaving the army, I got married and he was too. After 3 year of marriage, I saw him again. We drank together, remember all stories in the army then we had sex.

These sexual experiences can be seen in the context of increased opportunities for male–male sex as much as they are about reduced opportunities for sex with females. More recent examples of these closed settings include compulsory drug detention facilities and the added dimension of HIV risk because of the higher prevalence among people with a history of drug injecting. We purposively sampled current injecting drug users to also understand better the male-to-male sex experiences of men in these in closed settings. At the time the research for this paper was conducted the Ministry of Labor reported that in the decade to 2010, over 300,000 people had been detained in centres like these. They also reported that over 120 drug detention centres existed across Vietnam holding 40,000 people (Human Rights Watch, 2011). The increasing number of detoxification centres for people with drug (usually heroin) dependencies can be seen as a risk environment where men were in close proximity to each other for long periods of time. In one focus group for men who were current drug users, participants described a number of scenarios like the one below.

When I was in detox centre, I felt so lonely … My roommate is a pretty boy, and I asked myself why don't I ask him to have sex with me instead of masturbating. Later I acted on this thought … sometime after leaving the detox centre, I gradually lost my excitement with my wife … and these days, I often look for a man who has the appearance of a woman to have sex with…

Vietnam's changing economic landscape, increasing urbanisation and the sexualisation of public spaces by young people in Hanoi have been documented previously (Gammeltoft, 2002). These changing economic conditions also have implications for the increasing number of people moving from rural and regional areas to Vietnam's major cities. In our study, urbanisation and the development of a market economy and the changing nature of income generation opportunities could also be seen as explanations for why our participants reported being bisexually active.

I have been living in Hanoi for 8 years. When I first moved here my life is so difficult. I made friends with one man coming from central Vietnam. He has good job so could earn money and he helped me so much. He is a gay man. At first I had sex with him to repay him for his help, but over time our relationship got closer. Even after I married we often have sex with each other … He is my only male partner.

One time, I went out to Ha Le Lake where a man approached me while I was sitting alone … He offered me some money and asked me to have sex with him. I was a little scared but quite curious so I agreed. I needed money for life and honestly, this also makes me excited.

The lakes and parks nominated by our focus group participants as the most common spaces for meeting men and women are a ubiquitous feature of Hanoi. These environments have been documented as public spaces in which private encounters are commonplace (Gammeltoft, 2002). The changing urban and economic landscape in Hanoi also provides a number of privatised spaces around public places which could be viewed as potential risk environments for sexual activity.

## Limitations

The study has some limitations to consider when interpreting the findings. First, focus group participants were recruited through their contact with peer workers. Some men may also have been reluctant to participate in a focus group conducted at the chosen locations and this may have impacted on the key themes identified by the focus groups.

## Discussion

This study incorporates three different types of qualitative assessment of bisexual male behaviour in Hanoi, Vietnam and is one of the few in the GMS to do so (van Gemert et al., 2013). First, the study showed that behaviourally bisexually active men in our focus groups identified with a range of self-perceived sexual orientations. Second, the pairwise ranking found that behaviourally bisexual men met partners for sex in a diversity of places, the most common being parks and lakes for men and parks, lakes and also cafes for women. Third, the key findings highlighted the importance of understanding the spaces in which risk-taking occurs beyond a person's own health beliefs.

Comprehending how these different aspects intersect to describe the bisexually active male experience in Hanoi can be used to determine appropriate targeted public health interventions that will appeal to this population and be focussed on more than just information provision.

A key finding of this work is that 60% of the study participants (who were all behaviourally bisexual) indicated that they were either predominantly heterosexual or bisexual in their sexual orientation. This finding is consistent with prior studies that have also indicated that large proportion of MSM in Vietnam are bisexually active, and in most studies the majority of participants report themselves to be bisexual or heterosexual rather than homosexual (Colby, 2003; Toan et al., 2005). Taken together, these findings suggest that bisexual male behaviour in Hanoi is not only a consequence of homosexually oriented men having sex with women for reasons of societal constraint. Therefore health programmes which assume and focus on working with men of homosexual orientation may not be capturing some of the most pertinent players in terms of potential transmission of STIs including HIV. This highlights the need for a non-traditional and diverse focus in assessing the target audience for health promotion in this area.

Participants reported meeting partners for sex across a diversity of places with differences of choice depending on whether men were engaging in sex with other men or with women. Importantly, there were a range of public and private spaces, some of which like parks, reflected a degree of opportunistic interaction. The high frequency of park and lake meetings for both genders, but particularly for male–male encounters may be influenced by the degree of stigma associated with homosexual behaviour. Further work is required to understand how intimate encounters and risk are understood by bisexually active men, especially in the public spheres of parks and lakes. In addition, the rise of the internet and on-line social networking enables discretion in organising male–male sexual contact, as has already been documented amongst female sex workers offering more discrete services in Ho Chi Minh City (Kay Hoang, 2011).

The focus on these less-acknowledged spaces for sexual encounters implies that traditional approaches for distribution of sexual education messages (such as ‘saunas’ and massage parlours) would be unlikely to reach these behaviourally bisexual men. Similarly, the lack of defined physical spaces for such encounters means that practical programmes such as condom distribution may be challenging to deliver. There is a need for an increased understanding on the best ways to deliver health promotion programmes targeting bisexually active men across the variety of different spaces used for sexual negotiation by men in Hanoi. Better documentation and awareness of these risk environment spaces and how men themselves interpret risk in these spaces will also enable targeted delivery of these programmes.

Participants in this study also reported sexual experiences with other men in closed settings including drug detoxification centres and military or university dormitories. There is a need to recognise the heightened risk that may come from sexual behaviour between men in these spaces. As noted above, the development of public health messages in these settings requires further research and testing to ensure that it is targeted appropriately. The recent closure of many detention centres may even provide unintended benefits beyond the ethical and human rights rationale (Edington & Bayer, 2013).

Finally, the highlighted themes about male bisexual experiences point to issues facing Vietnam as an increasingly urbanising country with a developing market economy. These themes highlight the potential for the development and spread of STI in certain populations. For example, the traditional themes of marriage, acceptability of male–male sex outside of marriage and closed communities for young males contrast with newer themes of sexual income generation and the night life economy.

## Conclusions

As outlined and shown in other contexts (Volz, Frost, Rothenberg, & Meyers, 2010), bisexually active men have the potential to act as a bridge for sexually transmitted diseases between themselves and other members of the sexually active population in Vietnam. This is a concern as HIV rates amongst MSM are seemingly increasing faster than other risk populations in Vietnam.

The three different qualitative areas of this study all support the need to re-think the approaches for developing public health messages and programmes targeting bisexually active men in Vietnam. Responses require a realistic understanding of not only the diversity of spaces that are utilised in sexual interactions but also the pressure that increasing urbanisation brings to non-homosexual-identifying men engaging in bisexual sexual activity in Hanoi.

## Note

1. Countries of the Greater Mekong Sub-Region are Cambodia, China (Guangxi and Yunnan Provinces only), Lao PDR, Myanmar, Thailand and Vietnam.
